# Supplementary material for: Determinants of stunting in children under five years old in South Sulawesi and West Sulawesi Province: 2013 and 2018 Indonesian Basic Health Survey
Source: PLoS One. 2023 May 11;18(5):e0281962. doi: 10.1371/journal.pone.0281962 (PMC10174531; doi:10.1371/journal.pone.0281962)
Supplement: Attachment — Submitted filename: Response to Reviewers.docx [file pone.0281962.s001.docx]

Rebuttal letter in response to the editorial on reviewers' comments on the manuscript titled:

**Determinants of stunting in children under five years old in South Sulawesi and West Sulawesi Province: 2013 and 2018 Indonesian Basic Health Survey**

We thank the editor and the reviewers for the thoughtful inputs to our manuscript. In the revised version, we addressed all raised points. Please find below our response to each point the academic editor and reviewers raised. We hope that we clearly addressed all of them and that the manuscript will be now suited for publication.

Sincerely,

Hayani Anastasia

**Responses to editorial comments**

1. Please ensure that your manuscript meets PLOS ONE's style requirements, including those for file naming.

**Response:** *We have carefully reviewed the style manuscript and hope we meet PLOS ONE style requirements.*

2. Please amend the manuscript submission data (via Edit Submission) to include author Atmarita.

**Response:** *We will include the author Atmarita during revision submission*

3. We note that you have included the phrase "data not available" in your manuscript. Unfortunately, this does not meet our data sharing requirements. PLOS does not permit references to inaccessible data. We require that authors provide all relevant data within the paper, Supporting Information files, or in an acceptable, public repository. Please add a citation to support this phrase or upload the data that corresponds with these findings to a stable repository (such as Figshare or Dryad) and provide and URLs, DOIs, or accession numbers that may be used to access these data. Or, if the data are not a core part of the research being presented in your study, we ask that you remove the phrase that refers to these data.

**Response:** *We would like to change data availability to "The 2013 and 2018 Indonesian Basic Health Survey data used to support these study findings were supplied by the Health Development Policy Agency of the Indonesian Ministry of Health under license and so can not be made freely available. Requests for access to these data should be made to the Health Development Policy Agency of the Indonesian Ministry of Health (*[*http://www.badankebijakan.kemkes.go.id/layanan-permintaan-data/*](http://www.badankebijakan.kemkes.go.id/layanan-permintaan-data/)*; Em*ail: [datin.bkpk@kemkes.go.id](mailto:datin.bkpk@kemkes.go.id))

4. The two reviewers have pointed out important different issues for revision. In addition to the reviewer #1 comments on providing more methods details in the main text and prevalence with associated results in the abstract, I recomend to replace the statistical analyis using Prevalence Rates (it´s more apropriate for a cross-sectional study design with outcomes > 10% - please see the article by Barros & Hirakata. Alternatives for logistic regression in cross-sectional studies: an empirical comparison of models that directly estimate the prevalence ratio. <https://bmcmedresmethodol.biomedcentral.com/articles/10.1186/1471-2288-3-21>).

**Response:** *As suggested by the editor, we have replaced the statistical analysis using Prevalence Rates with Poisson Regression.*

**Responses to Reviewer 1**

Abstract

1. Are the areas districts, or provinces?

**Response**: The areas are provinces, and we have changed them accordingly in the manuscript

2. The study did not "try" but the study "determined"

**Response**: We thank the reviewer for the correction and we edited as below

“This paper aimed to determine the determinants of stunting among children under five in South and West Sulawesi Province using the 2013 and 2018 Indonesia Basic Health Survey.”

3. The title mentions 2013 and 2018, but abstract has 2007. Despite these two provinces having been one province and only divided in 2007, be clear about the years you considered for the data analyzed in this study. You are saying "This study used 2007, 2013, and 2018 Indonesian Basic Health Surveys"????

**Response**: We thank the reviewer for the correction and we edited the abstract.

4. Nowhere do you mention that the study was a retrospective/secondary data analysis? Was it not? No sample size is mentioned. In the results, mention the mean age of children. This is important. The study is about the under 5 year olds. Also, I am uncomfortable that the abstract has not statistical results, just a mention of the factors. The conclusion is not specific - needs to be revised. Since you have mentioned the households, maternal and child factors in the results, it is not necessary to repeat the sentence in a general manner like this in the conclusion. Linking the determinants with the recommendation is enough.

**Response**: We thank the reviewer for the suggestions and corrections and we edited the abstract.

Introduction

5. Should consider other countries since Plos One is an international platform with a broader readership. Mentioning global, and LICMs in general is good in paragraph 1. At least in the 3rd paragraph starting with "Studies reveal various household, parental, and child factors are associated with stunting"., briefly elaborate on their place of origin.

**Response**: We thank the reviewer for the suggestions and corrections. We elaborate on the place of the studies.

Method

6. It would interest the readers to read a brief description about the survey. Data came from a National survey, right? Does Indonesia has two provinces only - that description of Indonesia as a setting would be fair, until the readers get to understand that the two provinces; South Sulawesi and West Sulawesi were chosen based on what? Unless if they are the only provinces in Indonesia. Are they?

**Response**: We thank the reviewer for these comments and made amendments.

“South Sulawesi and West Sulawesi Province were chosen because of the high prevalence of stunting, and it was previously one province divided in 2007. The prevalence of stunting in West Sulawesi Province in 2018 was 41.59%, the second highest in Indonesia. At the same time, the prevalence of stunting in South Sulawesi was 35.74%, making it the fourth highest stunting in Indonesia.”

7. Much as you used previous data, it is still important to mention important aspects of the methods such as, sampling techniques used, sample size used in the national study. Also, what informed the extraction of which data to use in your study, especially on households, and maternal, for an example maternal age is missing in the variables you used to study the determinants. Except having to explain what informed selection of the variables you used, supposed some important variables like maternal age, weight, BMI were not there in the database, then, mention that as a limitation.
   Elaborating on the measurements and tools that yielded HAZ, as well as how were the characteristics of children and mother collected during 2013, and 2018 would improve on a paper.

**Response**: We thanked the reviewer for this questions. As suggested by the reviewer we made corrections on the method section

Result

8. Wellpresented. It is always good to differentiate the OR for unadjusted and adjusted models with COR (crude odds ratio) and AOR (adjusted odd ratio) respectively - subject to the authors.

**Response**: We thank the reviewer for the suggestions and corrections.

Discussion

9. Please revise these references and ensure that correct references reflective of the findings are added.

**Response**: We thank the reviewer for the suggestions and we have revised the references.

Strength and Limitation

10. Is there any limitation in using a retrospective data analysis, especially for data collected in 2007, or 2013 for an example? 2018 is still within 5 years at least. bias on variables analyzed?

**Response**: We thank the reviewer for the comment. We have addressed the limitation of this study especially on the selection of independent variables.

**Responses to Reviewer 1**

1. There are some sections, specially those dedicated to the results and the discussion, which textual fluidity is poor. There is a great amount of information but the sentences are a bit disconnected, without interpretation, demanding extra attention from the reader to understand the main message.

**Response**: We thank the reviewer for the comment. We rewrite the results and make some addition to the discussion. We hope it makes the manuscript more readable.

2. the aim of the study was “to compare the determinants of stunting among children under five years old in South and West Sulawesi as these two provinces were one province and only divided in 2007”. However, what was done in the text was listing the determinants in each area. There was no comparison between the results from each area and, as a consequence, the comparison itself was not subject of the discussion nor was mentioned in the conclusion.

**Response**: We thank the reviewer for the correction and we edited as below

“This paper aimed to determine the determinants of stunting among children under five in South and West Sulawesi Province using the 2013 and 2018 Indonesia Basic Health Survey.”

3. There are initials with no corresponding meanings (i.e. SSGI, IDD, e-PPGBM, ARI);

**Response**: We thank the reviewer for the correction and add what the abbreviation stands for.

4. There are some important methods description missing in the text. For example, there is no explanation about how variables were collected (interviews, anthropometric evaluation etc). Also, you do not describe which growth standards were used as reference to generate the z scores (World Health Organization?)

**Response**: We thank the reviewer for the suggestions and corrections. We made a change regarding the suggestion on method section as below”

“Two types of structured questionnaires were used: individual and household. In this analysis, we collected information from 10,862 women with children under five years old in South Sulawesi Province and 2,663 women with children under five years old in West Sulawesi Province…”

“Based on WHO growth standards, the height indicator for a period is determined based on the z-score or height deviation from average height. The WHO Anthro software was used to calculate the z-score. Stunted was categorized as 1 for stunted children and 0 for those not stunted. The limit for the nutritional status category according to the height index/age is: stunted: <-3.0 SD to -2.0 SD, normal: ≥ -2.0 SD”

5. Tables: the independent variables presented in the tables are a little bit confusing. For example, does the “mother’s employment” question refer to paid jobs? “Working” may include informal, formal and even domestic work. Does the “School” category mean that the mother is at school (i.e. student) or that she works in a school? Other details should be reviewed (mother’s height categories; questions to children under 5 years old such as “Have you ever been breastfed?”).

**Response**: We thank the reviewer for the suggestions and corrections. We made the change on the the tables.

6. The information contained in the figures 3 and 4 do not correspond to their title and/or subtitles.

**Response**: We thank the reviewer for corrections and agree with the correction. We deleted these figures because those are not correspond to the aim of this paper.

7. Some statements should be made with caution. At the end of the manuscript, for example, you mention “The limitation of our study was the use of cross-sectional data for a causal relationship”. However, it is important to remember that causal relationships can never be made with data from a cross sectional study. Therefore, you do not have a causal relationship.

**Response**: We thank the reviewer for the comment and agree with it.

**We thank the reviewers for their positive comments.**
